# Selectivity and ligand-based molecular modeling of an odorant-binding protein from the leaf beetle *Ambrostoma quadriimpressum* (Coleoptera: Chrysomelidae) in relation to habitat-related volatiles

**DOI:** 10.1038/s41598-017-15538-8

**Published:** 2017-11-13

**Authors:** Yinliang Wang, Yincan Jin, Qi Chen, Ming Wen, Hanbo Zhao, Hongxia Duan, Bingzhong Ren

**Affiliations:** 1 Jilin Provincial Key Laboratory of Animal Resource Conservation and Utilization, Northeast Normal University, Changchun, Jilin, China; 2 Department of Applied Chemistry, College of Science, China Agricultural University, Beijing, China; 3 Key Laboratory of Vegetation Ecology, MOE, Northeast Normal University, Changchun, China

## Abstract

In this study, the most abundant and antenna-specific odorant-binding protein (OBP) of the elm pest *A. quadriimpressum*, AquaOBP4, was expressed and purified. The selectivity of AquaOBP4 was investigated by screening against a panel of 40 habitat-relevant compounds. Based on the obtained results, a homologous model of AquaOBP4 was established. This model indicated that AquaOBP4 is highly homologous to DmelOBP LUSH and includes two main binding sites. A docking analysis showed that four of five active ligands bound at Site 1, whereas the other ligand was situated at Site 2. Furthermore, new ligands were docked in the model, and the results of fluorescence-based binding assays of these compounds were highly consistent with the binding conformation and binding affinity predicted by our model. Additionally, three binding odorants derived from elm leaves elicited a strong electroantennogram response and exerted a significant attractive effect on adult *A. quadriimpressum*. All of the results showed that AquaOBP4 is likely linked to the foraging behavior of *A. quadriimpressum*. This study provides a new reliable tool for future large-scale compound screenings and revealed several functional chemicals that might aid the development of a better pest management approach for *A. quadriimpressum*.

## Introduction

Environmental molecules contain information that regulates a series of important insect behaviors, such as mating^1,2^, oviposition^3^, foraging^4,5^ and host-seeking^6,7^. Insects have evolved a highly acute and sensitive olfactory system that can selectively detect environmental molecules. Several types of olfactory proteins play crucial roles in determining or helping to complete the selective detection process for odorants, including odorant-binding proteins (OBPs), odorant receptors (ORs), ionotropic receptors (IRs) and sensory neuron membrane proteins (SNMPs), along with odorant-degrading enzymes (ODEs)^8^. It is now commonly accepted that insect OBPs exhibit the function of solubilizing odorants to transport hydrophobic molecules through the aqueous sensilla lymph and contribute to the sensitivity of the olfactory system^9,10^.

Recently, several structures of insect OBPs, e.g., *Bombyx mori* PBP1 (BmorPBP1), *Anopheles gambiae* OBP1 (AgamOBP1), AgamOBP7, *Culex quinquefasciatus* OBP1 (CquiOBP1) and *Locusta migratoria* OBP1 (LmigOBP1), have been solved based on crystal structure, NMR and X-ray studies^1,11–15^. Structural analyses of BmorPBP1 led to the conclusion that residues Asp-132 and Glu-141 form a molecular switch that, at low pH, triggers the formation of the C-terminal α-helix upon protonation^16^ to aid in the binding and unlocking of the sex pheromone bombykol. This process is slightly different in mosquitoes, in which AgamOBP1 and CquiOBP1 lack an extended α-helix at the C terminus, and the hydrogen bond triad might be a pH-sensing lock that clamps the C terminus onto a bound odorant. At low pH, the hydrogen bond disrupts and unlocks the bound ligands^9,13^. In LmigOBP1, the seventh α-helix forms a wall in the form of an “L”-shaped internal hydrophobic cavity that accommodates linear ligands^15^. Most studies suggest that a pH-dependent conformational change is crucial for binding and releasing ligands^13,17,18^, although the binding pocket and conformational changes differ between different OBP-odorant complexes. For example, the C-terminal extensions of the *Anopheles gambiae* OBP1 (AgamOBP1), AgamOBP7 and *Culex quinquefasciatus* OBP1 (CquiOBP1) proteins are locked by a hydrogen bond triad composed of the last residue and two other residues (Tyr and His). The hydrogen bond triad is disrupted at lower pH levels, causing the C-terminal loop to shift away from the binding pocket and the to “lid” open. In the silkworm, *Bombyx mori*, the C-terminal dodecapeptide segment of PBP (BmorPBP) forms an additional helix in the protein core, occupies the corresponding pheromone-binding site and releases ligands at lower pH levels^11,13,14^.

Many studies show that OBPs exhibit a preliminary selection function for odorants, acting as a first step in olfactory sensing in insects^19–21^. Considering this role as the first level of olfactory ligand recognition, structural and functional studies of OBPs will not only give us a better understanding of insect peripheral olfaction detection but also help us design more effective structure-based pest management compounds. The most elegant method for calculating the binding affinities of active odorants with OBPs is to express and purify recombinant proteins in an *E. coli* system and test *in vitro* fluorescence quenching resulting from the replacement of a reporter (usually 1-NPN)^22,23^. However, due to the large amount of work required to obtain pure proteins, the capacity to screen for insect OBPs is still limited. Furthermore, when testing binding affinities, odorants are usually randomly selected, or physiologically relevant compounds are used as a reference^24^; thus, there is no preliminary screening method for the massive pool of candidate compounds. Therefore, an effective and reliable guiding tool is needed for large-scale compound screening.

The beetle *Ambrostoma quadriimpressum* (Coleoptera: Chrysomelidae), a major forest pest, is a monophagous species found in the East Asian region, particularly in northeast China, where it feeds exclusively on the shoots and leaves of elms (*Ulmus pumila*, *Ulmus macrocarpa* and *Ulmus japonica*), leading the tree to suffer irregular death before it is fully grown and causing great damage to the timber industry as well as city afforestation^25^. In previous RNA sequencing (RNA-Seq) studies, we identified fifteen candidate OBPs with complete ORFs in this species and determined that AquaOBP4 was particularly abundant in the antennae compared with other AquaOBPs as well as highly antenna specific^26^. To explore the function and selectivity of AquaOBP4, we attempted to express and purify this protein and to screen for active odorants associated with it. Furthermore, we aimed to establish a reliable molecular screening model using the obtained data to provide a better understanding of the interaction between OBP structure and active odorants, which will be useful for large-scale ligand screening in the future.

## Materials and Methods

### Insects

The *A. quadriimpressum* population used in this study was initially obtained from elm (Ulmus pumila) trees in Jilin province of China in 2014 (E125.3 N43.9, E124.2 N 44.5). To maintain the maximum population of insects of the same age, we collected the insects within a small range in early June, which is the peak period for newly emerged adults every year. The adults of *A. quadriimpressum* were maintained at 28 °C and 85% RH under a 12:12 h light:dark cycle in a BIC-300 artificial climate chest (Boxun, Shanghai, China) immediately after collection. The population was fed fresh elm leaves. The population size of adults was greater than three hundred, with a male/female ratio of approximately 1:1. The sample collection was authorized by the forestry bureau of Jilin province. All experiments (RNA extraction, Y-tube and EAG assays) were performed three to five days after the collection of adults from the field, which were maintained as described above.

### RNA extraction and cDNA synthesis

The antennae of *A. quadriimpressum* females were carefully separated with diethyl pyrocarbonate (DEPC)-treated forceps under a stereomicroscope (Motic, Hong Kong, China). Tissues were collected from 60 individuals (at a male/female ratio of 1:1). The collected tissues were stored on ice in a DEPC-treated 1:1 water/ethanol solution until use. The total RNA from homogenized antennae was isolated using the TRIzol reagent (Invitrogen, Carlsbad, CA, USA) according to the manufacturer’s protocol and then subjected to ethanol/isopropanol precipitation in a final solution with 30 μl of RNase-free water. After extraction, the total RNA was quantified with a NanoDrop 2000 (Thermo Fisher Scientific, Waltham, MA, USA) and in a 1% agarose gel. The UV absorption values at 230 nm/260 nm and 260 nm/280 nm were recorded to monitor the purity of the RNA products, and the mRNA smear above the 28S rRNA band was checked to verify RNA integrity. The total antenna RNA was transcribed into cDNA in a reaction (total volume of 20 μl) containing 4 μl of first-strand buffer (250 mM Tris pH 8.3, 375 mM KCl, and 15 mM MgCl_2_), 1 μl of 10 mM dNTP mix, 1 μl of RNaseOUT, 1 μl of DTT (0.1 M), 1 μl of an oligo-(dT) 20 primer (50 μM) and 1 μl of Superscript III reverse transcriptase (200 units/μl) (Invitrogen, Carlsbad, CA, USA). The cDNA synthesis program consisted of 45 min at 50 °C followed by 15 min at 70 °C.

### AquaOBP4 cloning

Based on previous RNA-Seq analysis, fifteen candidate OBPs with complete ORFs were identified in *A. quadriimpressum*. AquaOBP4 was shown to be the most abundant and antenna-specific OBP among all of the AquaOBPs identified (Table 1). To explore the function and selectivity of AquaOBP4, we attempted to express and purify this protein and screen for active odorants that associated with it. Primers were designed based on RNA-Seq data, and the designed primers amplified complete 145-aa AquaOBP4 sequences from the antennal cDNA of *A. quadriimpressum* using PrimeSTAR Max DNA Polymerase (TaKaRa, Japan), which resulted in removal of the signal peptide sequence (AquaOBP4 sense: 5′-ATGAACGAGAAACAAATGGA-3′; AquaOBP4 antisense: 5′-TCAATGCAGAAAAAATTTCTC-3′). The PCR thermocycling conditions were as follows: 1 min 30 s at 94 °C followed by 32 cycles of 94 °C for 1 min 30 s, 53 °C for 30 s and 68 °C for 30 s and a final extension of 7 min at 68 °C. The PCR products were run in 1% agarose gels and visualized with Goldview Nucleic Acid Stain (Dingguo, China). The DNA bands with the expected lengths were cut, gel purified with the MiniPrep Gel Extraction Kit (Sangon, China) and cloned into the cloning vector (pUCm-T vector system; Sangon, China). The cloned PCR products were analyzed in an ABI 3730xl DNA Analyzer sequencing system using vector primers. After verifying that the sequence was correct, a 6X-His tag was added to the sense primer for purification when it was subcloned to the pET28a vector using the restriction sites Nco I and BamH I.

**Table 1:** Detailed information on the candidate AquaOBPs of *A. quadriimpressum*.

Gene name	Gene ID	ORF (aa)	Status	Isoelectric point	FPKM (Antenna/Leg)
AquaOBP 1	EFA05677.1	126	Complete ORF	4.91	326.34/3.01
AquaOBP C1	EEZ97789.1	128	Complete ORF	4.93	87.41/1.59
AquaOBP 2	EFA05677.1	125	Complete ORF	4.92	22.75/0.61
AquaOBP C2	ADD82417.1	169	Complete ORF	5.78	10.71/36.01
AquaOBP 3	EFA10713.1	142	Complete ORF	8.80	1.25/0.49
AquaOBP C3	ADD82417.1	137	Complete ORF	5.29	0.55/190.4
AquaOBP 4	EFA10713.1	145	Complete ORF	8.01	1225.47/1.8
AquaOBP C4	ADD82417.1	134	Complete ORF	6.43	0.83/33.47
AquaOBP 5	EFA05695.1	250	Complete ORF	5.39	2.94/3.3
AquaOBP C5	ADD70031.1	135	Complete ORF	5.66	185.9/1.96
AquaOBP 6	EFA02960.1	150	Complete ORF	4.69	134.95/172.13
AquaOBP C6	AGH70097.1	150	Complete ORF	4.94	184.45/105.61
AquaOBP 7	XP_008200270.1	153	Complete ORF	9.37	38.38/1.23
AquaOBP C7	EFA01425.1	119	Complete ORF	8.39	0.02/0.75
AquaOBP 8	ADG96060.1	152	Complete ORF	5.78	0.46/0

### AquaOBP4 expression and purification

The positive clone verified by DNA sequencing was inoculated in 5 mL of LB medium with ampicillin (50 μg/mL) and incubated at 225 rpm at 37 °C for 4 h. Thus, culture was then diluted into 2 L of LB medium, and the cells were grown until the optical density at 600 nm (OD600) reached 0.6. Next, 0.06 mM isopropyl-beta D-thiogalactopyranoside (IPTG) was added, and expression was induced for 2 h at 37 °C, after which the cultures were grown at 15 °C for 12 h. The cells were subsequently collected, and proteins in the periplasmic fraction were extracted with buffer A (20 mM Tris-HCl, 300 mM NaCl, and 1% Triton-100, pH 8.0) using ultrasonication. After centrifugation of the sample at 16,000 × g, the precipitate was dissolved in buffer B (20 mM Tris-HCl, 300 mM NaCl, 1% Triton-100, and 8 M urea, pH 8.0) and loaded onto a Ni-IDA column (BioVision, USA). The column was then washed using 100 ml of buffer C (20 mM Tris-HCl pH 8.0, 2 M NaCl, 0.1% TritonX-100, and 8 M urea) and 50 ml of buffer D (20 mM Tris-HCl pH 8.0, 50 mM NaCl, 0.1% TritonX-100, 10 mM imidazolium, and 8 M urea) to remove impurities, enzymes and non-target proteins. The target proteins were subsequently washed and collected using 50 ml buffer E (20 mM Tris-HCl pH 8.0, 50 mM NaCl, 0.1% TritonX-100, 250 mM imidazolium, and 8 M urea), and the denatured proteins were renatured via dialysis in a gradient of buffer F (50 mM Tris, 50 mM NaCl, 0–6 M urea, 5 mM GSSG, 2 mM GSH, and 1 mM DTT, pH 8.0) (Fig. S1). The 6X-His tag was subsequently cut using thrombin (Ye Sen, China), and the protein was reloaded on a Ni-IDA column to further remove His tag-labeled proteins. The final protein product was dissolved in 20 mM Tris-HCl (pH 8) at a concentration of 1.2 mg/ml for further testing.

### Fluorescence binding assays

Binding affinity was primarily tested with a panel of 40 *A. quadriimpressum* habitat-related compounds, most of which are abundantly distributed in elms and *A. quadriimpressum*. Additionally, one newly identified repellent, cinnamaldehyde, was included^25,27,28^. After the molecular model of AquaOBP4 was established, we selected two additional compounds, which also came from elms and *A. quadriimpressum*, for model validation. All of the compounds were purchased from Sigma-Aldrich (Millipore Sigma, USA) with purities of greater than 95%. The CAS numbers, structures and sources of the tested compounds are listed in Table 2, and detailed information (including content) for each odorant is provided in Table S1.

**Table 2:** Detailed information for the compounds for testing against AquaOBP4.

Name	CAS No.	Source	Purity	Molecular Structure
Tested Compounds				
1-(4-Ethylphenyl)-ethanone	937-30-4	Leaves of elm	97%	
Methyl benzoate	93-58-3	Leaves of elm	99%	
cis-3-Hexen-1-ol	928-96-1	Leaves of elm	≥98%	
1-Methylnaphthalene	90-12-0	Leaves of elm	≥95%	
Caryophyllene	87-44-5	Leaves of elm	≥98.5%	
Dibutyl phthalate	84-74-2	Leaves of elm	99%	
Linalool	78-70-6	Leaves of elm	97%	
Nerolidol	7212-44-4	Leaves of elm	98%	
Pentadecane	629-62-9	Leaves of elm	≥99%	
Tetradecane	629-59-4	Leaves of elm	≥99%	
Limonene	5989-27-5	Leaves of elm	97%	
α-Farnesene	502-61-4	Leaves of elm	98%	
α- Pinene	7785-70-8	Leaves of elm	≥99%	
1-Tridecene	2437-56-1	Leaves of elm	96%	
Nonanal	124-19-6	Leaves of elm	95%	
Methyl salicylate	119-36-8	Leaves of elm	≥99%	
Dodecane	112-40-3	Leaves of elm	≥99%	
Benzaldehyde	100-52-7	Leaves of elm	≥99.5%	
trans-2-Hexenal	6728-26-3	Leaves of elm	98%	
3,7-Dimethyl-1,3,6-octatriene, ocimene	13877-91-3	Leaves of elm	≥90%	
Diisobutyl adipate	141-04-8	Leaves of elm	99%	
Leaf acetate	3681-71-8	Leaves of elm	≥98%	
Diisobutyl phthalate	84-69-5	Leaves of elm	99%	
Benzyl benzoate	120-51-4	Leaves of elm	≥99.0%	
3′,4′-Dimethylacetophenone	3637-01-2	Leaves of elm	98%	
Phenylacetaldehyde	122-78-1	Cuticle of larvae	≥95%	
Acetic acid, Phenyl ester	122-79-2	Cuticle of larvae	99%	
Hexanal	66-25-1	Phloem of elm trees	98%	
2-Heptanone	110-43-0	Phloem of elm trees	≥98%	
Myrcene	123-35-3	Phloem of elm trees	≥95%	
α-Terpinen	99-86-5	Phloem of elm trees	≥95%	
Heptadecane	629-78-7	Phloem of elm trees	99%	
Camphene	79-92-5	Adult feces	95%	
Indole	120-72-9	Adult feces	≥99%	
(+)-Cedrol	77-53-2	Adult feces	≥99%	
β-Ionone	14901-07-6	Adult feces	96%	
cis-Jasmone	488-10-8	Adult feces	90%	
cis-3-Hexenyl benzoate	25152-85-6	Adult feces	≥97%	
Ethyl palmitate	628-97-7	Adult feces	≥99%	
Cinnamaldehyde	104-55-2	Oak trees	99%	

N-phenyl-1-naphthylamine (1-NPN) was used as a reporter to measure the affinity of the fluorescent 1-NPN ligand to proteins^29^. The fluorescence spectra were recorded using a Fluoromax-4 spectrofluorometer (HORIBA Jobin Yvon, New York, USA) with 2 µM protein in 50 mM ammonium acetate (pH 7.2) at room temperature; the compounds and 1-NPN were dissolved in high-performance liquid chromatography (HPLC)-grade methanol at a concentration of 1 mM. Parameter selection was such that the slit widths for both excitation and emission were 10 nm. The reporter was excited at 337 nm, and the emission spectra were recorded between 380 and 500 nm. The AquaOBP4 saturation curve was tested using 1-NPN at 2 to 14 µM, and the affinities of the compounds were estimated using 2 µM 1-NPN and 10 to 70 µM concentrations of the compounds. The curves were then linearized using Scatchard plots. IC50 and Kd values were analyzed using Prism 6 (GraphPad Software, USA), and Ki values were calculated using the following equation: Ki = [IC50]/1 + [1-NPN]/Kd, where [1-NPN] is the concentration of dissociative 1-NPN and Kd is the dissociation constant of the protein/1-NPN complex. To investigate the response of AquaOBP4 at low pH, the fluorescence binding affinity between AquaOBP4 and one of the best ligands was measured at pH 5. Then, 50 mM ammonium acetate (pH 5) was used as a buffer to dissolve the AquaOBP4 protein.

### Molecular modeling of AquaOBP4

The modeled structures of AquaOBP4 were obtained using a template of DmelOBP LUSH (1OOH) employing on-line Swiss-model software^30^. The molecular conformations of all ligands were constructed in Sketch mode and optimized using the Tripos force field and Gasteiger-Hückel charge. The Surflex-Dock algorithm of SYBYL 7.3 was employed for the molecular docking study^31^. The binding cavity was set as “Auto”, and the Total Score was used to evaluate the binding affinity between the ligand and protein^32^. All molecular modeling predictions between the putative AquaOBP4 protein and ligand were conducted using the Silicon Graphics® (SGI) Fuel Workstation (Silicon Graphics International Corp., CA, USA).

### Electroantennogram responses of binding odorants

To measure the electrophysiological responses of the three best-binding ligands, including dibutyl phthalate, diisobutyl phthalate and butyl octyl phthalate, we used EAG assays^33^. In brief, glass electrodes fabricated with a PC-10 micropipette puller (Narishige, To kyo, Japan) were filled with a solution of 1 M potassium chloride and 1% polyvinylpyrrolidone. The reference electrode was then inserted into the eye of an *A. quadriimpressum* individual, and the recording electrode was placed at the truncated tips of the antennae using an MP-12 micromanipulator (Syntech, Kirchzarten, Germany). EAG signals were acquired with a high-impedance AC/DC pre-amplifier, and the data were analyzed with EAG Pro version 2.0 software (Syntech, Kirchzarten, Germany). A syringe cylinder was used as a cartridge to deliver 20 μl of odorant solution in paraffin oil at the desired dose (from lowest to highest). The odorant was loaded on a filter paper strip, which was then placed in a disposable syringe cylinder that served as the cartridge. The odorants were delivered into a consistent airstream (the flow rate was 500 ml/min, and humidity was 60–70%) at an approximate 1-cm distance from the antenna through a 20-G needle. The pulse duration was 0.1 s, and the recording time was set for 4 s, with a 2-min gap between stimuli for the recovery of EAG sensitivity. At least six individuals (three males and three females) were tested for each replicate at concentrations of 0.1, 1, and 10 µg/µl. Negative controls (paraffin oil) were performed first for each preparation, and the EAG peaks of each individual were normalized to the negative control.

### Y-tube olfactometer

**Figure 1:**
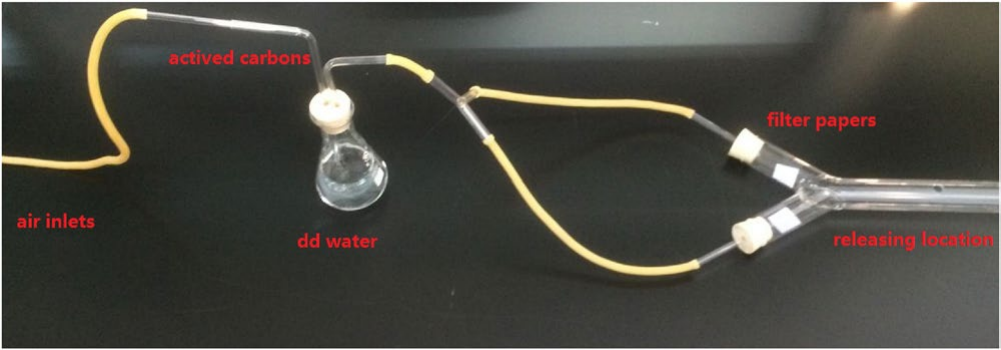
Experimental setup for the Y-tube olfactometer. Y-tube (1 cm diameter, 100 cm base length, 10 cm arm length). One small hole was cut into the base of the Y-tube at 10 cm for beetle release. For this experiment, a piece of filter paper (25 × 25 mm) was placed in both arms to test the odorants, and the beetles were then released from the hole at a 10 cm distance.

The responses of *A. quadriimpressum* to the three best ligands were assessed in a Y-tube olfactometer. The compounds were tested at an intermediate concentration of 10 µg/µl in paraffin oil. In these assays, 20 μl of the odorant was applied to a piece of filter paper (25 × 25 mm), which was then placed in one of the olfactometer arms. In the other arm, 20 μl of paraffin oil was used as a negative control. Thirty beetles (15 males and 15 females) were individually tested, with three replicates for each individual (for a total of 90 replicates). A red light source was used in the darkroom to avoid light interference. For the Y-tube (1-cm diameter, 100-cm base length, 10-cm arm length), one small hole was cut into the base of the Y-tube at 10 cm for releasing beetles. The source of the air that passed through the Y-tube was a pressurized tank of pure air. The air was first filtered through active carbon and then humidified by bubbling it through a bottle filled with double-distilled (dd) water before entering the Y-tube. The air flow traveled through both arms of the Y-tube olfactometer at a speed of 300 ml/min. Each individual beetle was placed at the entrance of the olfactometer (Fig. 1), and their “choice” was recorded when the beetle entered an arm and remained there for 30 s. If an insect made no choice within 3 min, the result was recorded as “no-choice”. The Y-tube olfactometer was cleaned with dehydrated alcohol and allowed to air-dry between trials involving different treatments or odorant dilutions. The positions of the odor sources were exchanged after every 10 beetles. The number of choice insects was used to show the repellency/attraction of odorants in different treatments, and significant differences were tested via two-way ANOVA.

## Results

### AquaOBP4 cloning and expression

Based on previous RNA-Seq data, AquaOBP4 showed the highest expression level among all of the 15 identified candidate AquaOBPs. Additionally, AquaOBP4 exhibited the highest antenna-specific value, with an antenna/leg expression ratio of 680.82 (Table 1). Thus, we selected this protein for further binding and modeling studies. AquaOBP4 cloning yielded a 139-aa protein product, including start and stop codons, with six conserved cysteines and typical characteristics of classical OBPs. The results of recombinant pET28a-AquaOBP4 vector sequencing were consistent with the RNA-Seq data, which confirmed our previous results. The predicted MW of AquaOBP4 was 16.06 kDa, and the theoretical pI was 8.31, with a 21-aa signal peptide (http://web.expasy.org/compute_pi and http://www.cbs.dtu.dk/services/SignalP). Based on the results of the first attempt at small-scale expression, we optimized the induced expression conditions to 0.06 mM IPTG and 15 °C for 12 h. After ultrasonication, most of the target proteins were found in the form of inclusion body. Therefore, we decided to purify the AquaOBP4 protein under denaturing conditions. To maintain the natural structure of AquaOBP4 after expression and purification, the target protein was renatured, and the 6X-His tag was cut off. Finally, we obtained 18 mg of the target protein, with a purity of 95% for further study (Grab-IT version 2.5, Japan).

AquaOBP4 was observed to be the most abundant OBP in the antenna of *A. quadriimpressum*, and its expression level was notably higher than that of any other AquaOBP, with a fragments per kilobase of transcript per million mapped reads (FPKM) value of 1225.47. Predictions revealed that AquaOBP4 is an alkaline protein with a pI of 8.31 and a 21-aa signal peptide.

### Binding assays

**Figure 2:**
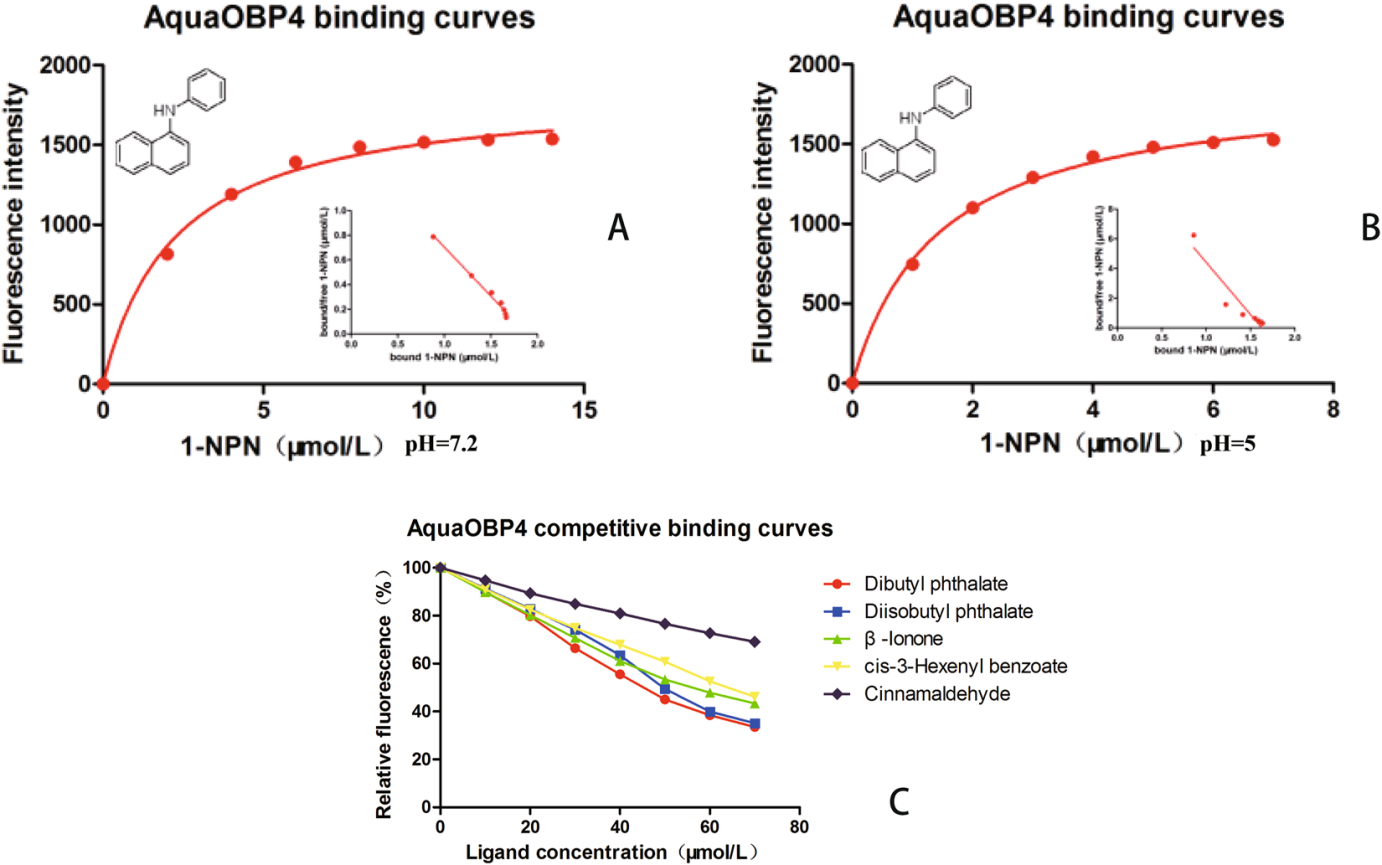
Binding curves of AquaOBP4 with the fluorescence reporter and active odorants. (**A**) Saturation curve of AquaOBP4 with the fluorescence reporter 1-NPN at pH = 7.2; The X axis ranged from 0–15 μM; a Scatchard plot is inserted under the curves. (**B**) Saturation curve of AquaOBP4 with the fluorescence reporter 1-NPN at pH = 5.0; The X axis ranged from 0–8 μM; a Scatchard plot is inserted under the curves. (**C**) Binding curves of five active odorants that bind to AquaOBP4; red: dibutyl phthalate, purple: diisobutyl phthalate, green: β-ionone, yellow: cis-3-hexenyl benzoate; gray: cinnamaldehyde.

The binding affinity of the 1-NPN fluorescence reporter to AquaOBP4 was tested first, and the Kd value of AquaOBP4 was shown to equal 2.27 ± 0.16 μM at pH 7.2 (Fig. 2A). Further tests on 40 *A. quadriimpressum* habitat-related compounds showed that most of the candidate ligands presented no 1-NPN displacement whatsoever. However, five compounds showed 1-NPN displacement. After preliminary screening, five compounds with low binding levels were chosen to evaluate their dissociation constants, including dibutyl phthalate, diisobutyl phthalate, β-ionone, cis-3-hexenyl benzoate and cinnamaldehyde. Dibutyl phthalate and diisobutyl phthalate occur in the leaves of elm trees, and β-ionone and cis-3-hexenyl benzoate came from the feces of adult *A. quadriimpressum*. Cinnamaldehyde, which was recently identified as a strong repellent of *A. quadriimpressum*, was also detected. These compounds were tested at final concentrations of 10 to 70 μM to evaluate their dose-dependent effect. The results indicated that the repellent, cinnamaldehyde, showed weak binding to AquaOBP4, with a dissociation constant of 79.26 μM. The other four compounds were ranked as follows in order of the highest binding affinity: dibutyl phthalate (Ki = 24.22 μM), diisobutyl phthalate (Ki = 26.77 μM), β-ionone (Ki = 30.06 μM) and cis-3-hexenyl benzoate (Ki = 35.00 μM) (Table 3 and Fig. 2B).

**Table 3:** Binding affinities of AquaOBP4 to tested compounds.

Ligands	IC50	Ki	Ligands	IC50	Ki
1-(4-Ethylphenyl)-ethanone	—	—	Nonanal	—	—
Methyl benzoate	—	—	Methyl salicylate	—	—
1-Methylnaphthalene	—	—	Dodecane	—	—
Caryophyllene	—	—	Benzaldehyde	—	—
Dibutyl phthalate	45.57	24.22	trans-2-Octenal	—	—
Linalool	—	—	3,7-Dimethyl-1,3,6-octatriene, ocimene	—	—
Nerolidol	—	—	cis-3-Hexen-1-ol	—	—
Pentadecane	—	—	Diisobutyl adipate	—	—
Tetradecane	—	—	Leaf acetate	—	—
Limonene	—	—	Diisobutyl phthalate	50.36	26.77
α-Farnesene	—	—	Benzyl benzoate	—	—
α-Copaene	—	—	1-Tridecene	—	—
Phenylacetaldehyde	—	—	Acetic acid, phenyl ester	—	—
3′,4′-Dimethylacetophenone	—	—	Hexanal	—	—
Myrcene	—	—	2-Heptanone	—	—
Heptadecane	—	—	α-Terpinen	—	—
Indole	—	—	Camphene	—	—
β-Ionone	56.56	30.06	(+)-Cedrol	—	—
cis-3-Hexenyl benzoate	65.85	35.00	cis-Jasmone	—	—
Cinnamaldehyde	149.1	79.26	Ethyl palmitate	—	—

The fluorescence binding assays showed that most of the tested compounds did not bind to AquaOBP4 in the first-round tests, although five ligands presented weak binding. Compared with the binding affinities of compounds from insects of other taxa, such as CquiOBP1 and AgamOBP1, the binding affinity of AquaOBP4 was relatively low^9,13^, and the results suggested that the best ligands for AquaOBP4 might still not have been identified at this stage. Therefore, we decided to establish a reliable homologous model of AquaOBP4 for predicting the active odorants.

### 3D model of AquaOBP4

**Figure 3:**
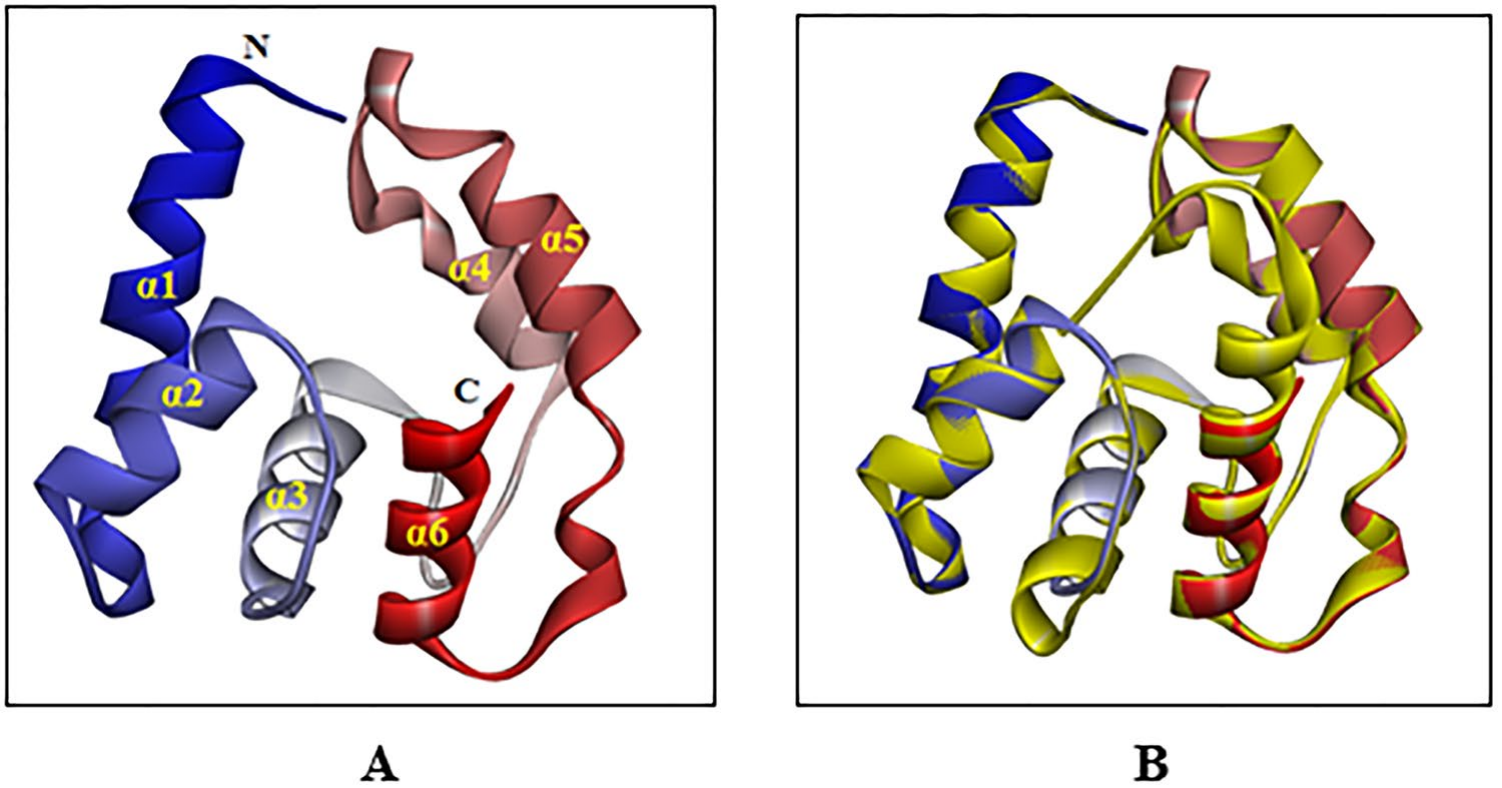
(**A**) The predicted 3D model of AquaOBP4 based on the crystal structure of DmelOBP LUSH. (**B**) The alignment plot of the targeted protein AquaOBP4 and the template protein LUSH (yellow, ID: 1OOH).

Because the sequence identity between the target protein AquaOBP4 and the template protein DmelOBP LUSH was 31.82%, a 3D model of AquaOBP4 was reasonably constructed based on the crystal structure of DmelOBP LUSH. The AquaOBP4 structure exhibited the overall folding of “classical OBPs” and was mostly helical (Fig. 3A). The 3D structure of AquaOBP4 included six α-helices located between residues Ser23-Lys46 (α1), Thr48-Asp59 (α2), Ala66-Leu79 (α3), Asn87-Pro99 (α4), Asp100-Lys114 (α5), and Asp115-Met134 (α6). Additionally, the 3D structures of the targeted protein AquaOBP4 and the templated protein DmelOBP LUSH aligned well with each other (Fig. 3B); hence, the putative model of AquaOBP4 was successfully used for exploring the binding conformation and binding affinities between AquaOBP4 and ligands.

### Molecular docking

**Figure 4:**
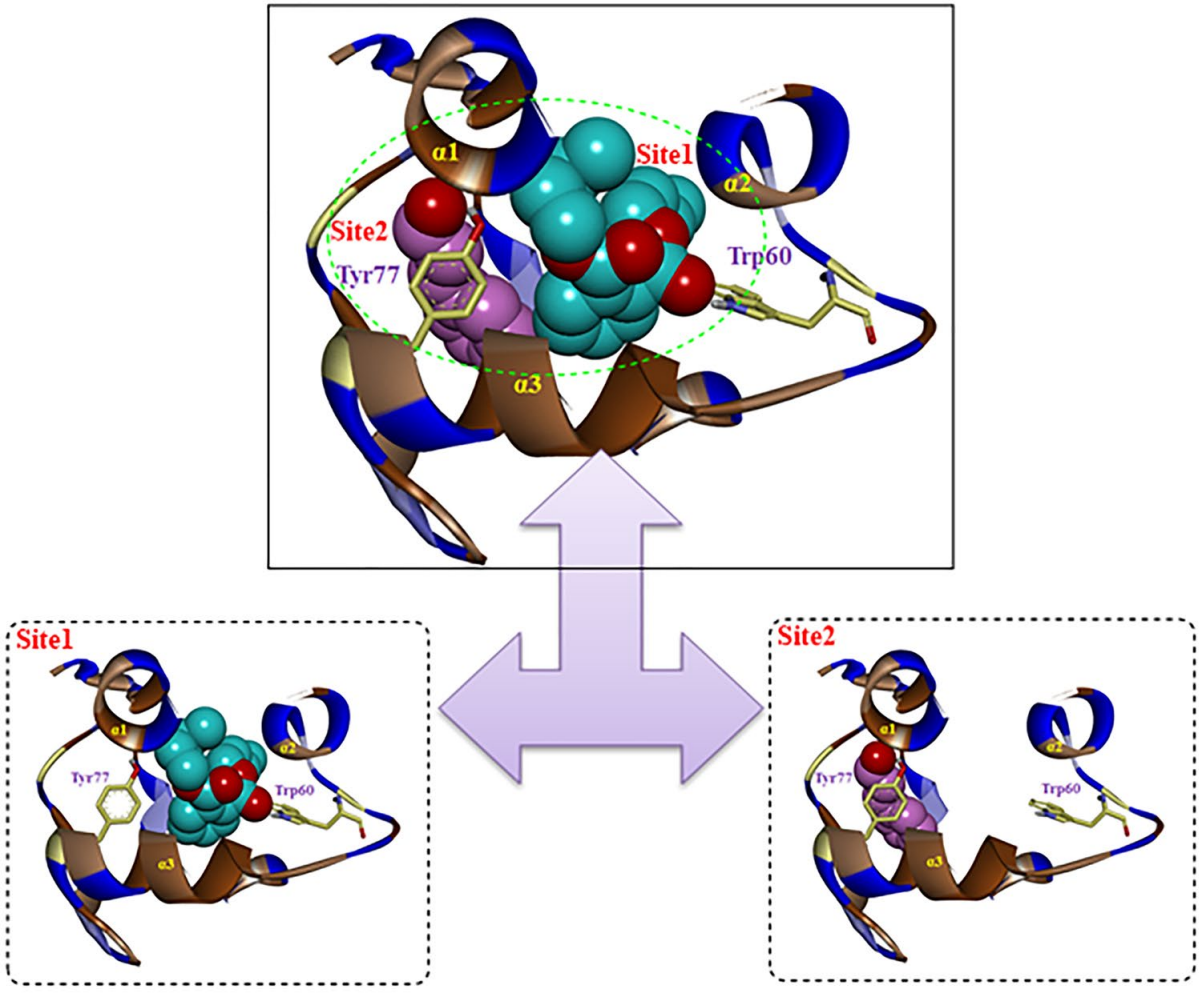
Two different binding sites, Site 1 (cyan) and Site 2 (pink), for ligands in AquaOBP4. A ligand is indicated in CPK mode with different colors of Dibutyl phthalate (cyan) and cinnamaldehyde (pink). H-bond residues of putative AquaOBP4 are indicated as yellow sticks. Hydrophobic residues of putative AquaOBP4 are indicated as a brown helix.

As shown in Fig. 4, the docking results for all ligands indicated that there were two different sites in the binding cavity of AquaOBP4. Site 1 was mainly a central binding region between helices α1, α2 and α3 and was surrounded by the unique, long, narrow hydrophobic helix α3. However, Site 2, located to the left of Site 1, far from the center region, was closely positioned behind helices α4 and α5 in AquaOBP4.

Four ligands, including dibutyl phthalate, diisobutyl phthalate, β-ionone, and cis-3-hexenyl benzoate, aligned well according to the docking results and were almost located at the same position as Site 1 of the AquaOBP4 model. Another ligand, cinnamaldehyde, was positioned at Site 2 (Fig. 4), far from Site 1 in AquaOBP4. As shown in Table 4, the docking scores of these four ligands were much higher than that of cinnamaldehyde, which agreed with the results of the binding affinity experiment.

**Table 4:** Structures, binding affinities and binding scores of five ligands in the training set and two ligands in the test set for AquaOBP4.

No.	Name	Structure	IC_50_	K_i_(μM)	pKi	Score
1	Dibutyl phthalate		45.57	24.23	4.62	6.38
2	Diisobutyl phthalate		50.36	26.77	4.57	6.01
3	β-Ionone		56.56	30.07	4.52	5.58
4	Cis-3-Hexenyl benzoate		65.85	35.01	4.46	4.80
5	Cinnamaldehyde		149.1	79.26	4.10	3.75
6^*^	Butyl octyl phthalate		23.89	12.70	4.90	8.02
7^*^	Isoeugenol		320.8	170.54	3.77	3.86

**Figure 5:**
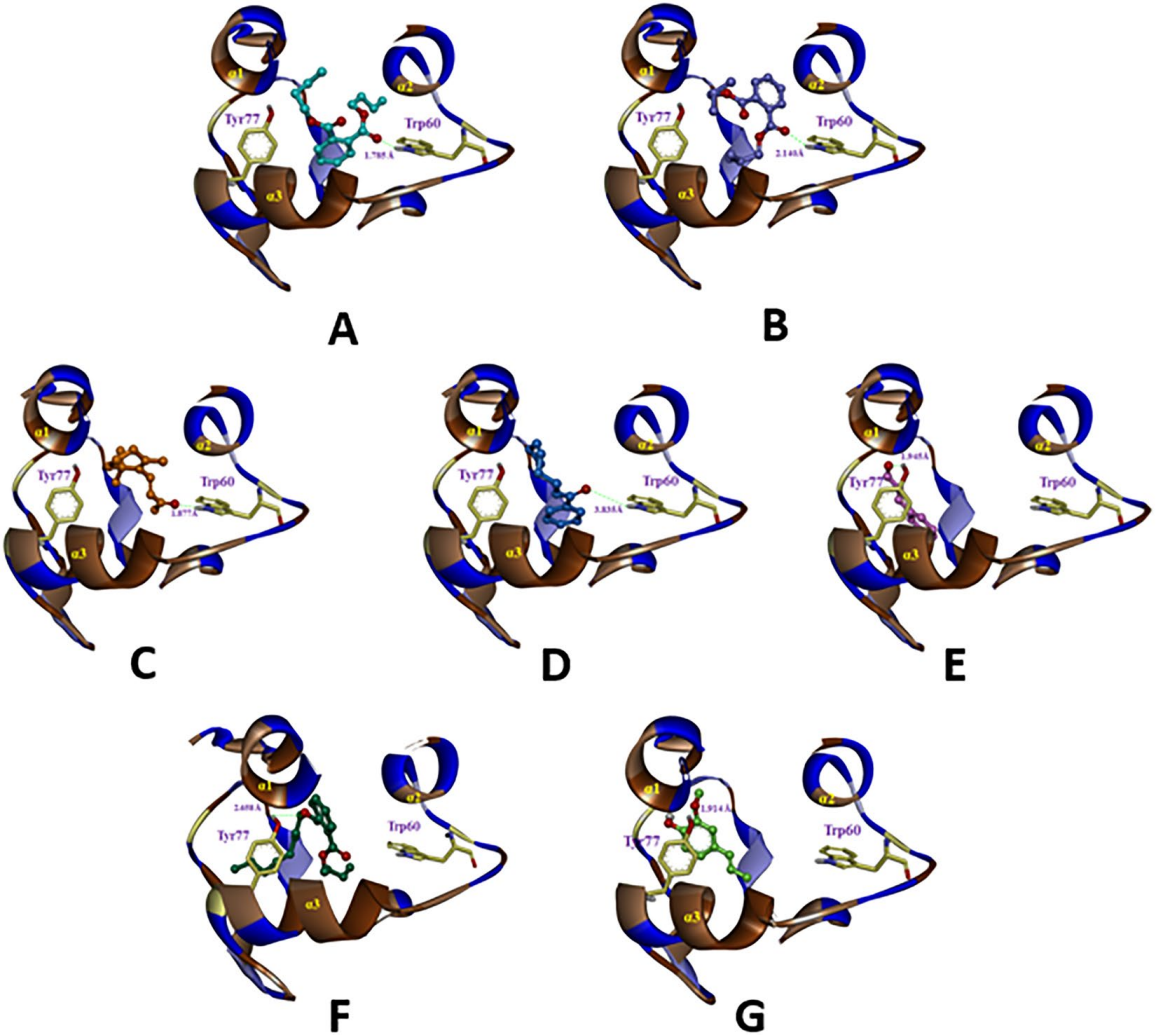
The binding modes of seven active odorants. The binding modes of (**A**) dibutyl phthalate (cyan), (**B**) diisobutyl phthalate (purple), (**C**) β-ionone (orange), (**D**) cis-3-hexenyl benzoate (blue), (**E**) Cinnamaldehyde (pink), (**F**) butyl octyl phthalate (dark green) and (**G**) isoeugenol (light green) in AquaOBP4.

To further clarify the binding conformation of ligands in AquaOBP4, the binding conformations of all ligands are shown in Fig. 5. The results showed almost the same H-bonding interactions between different O atoms of four ligands with high binding affinities and the key residue Trp60 of helix α2, with distances of 1.785, 2.140, 1.877, and 3.835 Å. It was clear that the predicted binding cavity for the four ligands in Site 1 was a visible hydrophobic region surrounded by three helixes, namely, α1, α2, and α3 (shown in brown), in AquaOBP4. Trp60 was indicated as an important residue for the location and orientation of these ligands in the binding pocket of AquaOBP4.

However, the low binding capacity of cinnamaldehyde alone revealed another H-bond with the Tyr77 residue in helix α3 of Site 2, with a distance of 1.945 Å, indicating that its location was far from the central binding region at Site 1 of AquaOBP4 and yielding a low binding score of 3.85.

### Further validation of the docking model

**Figure 6:**
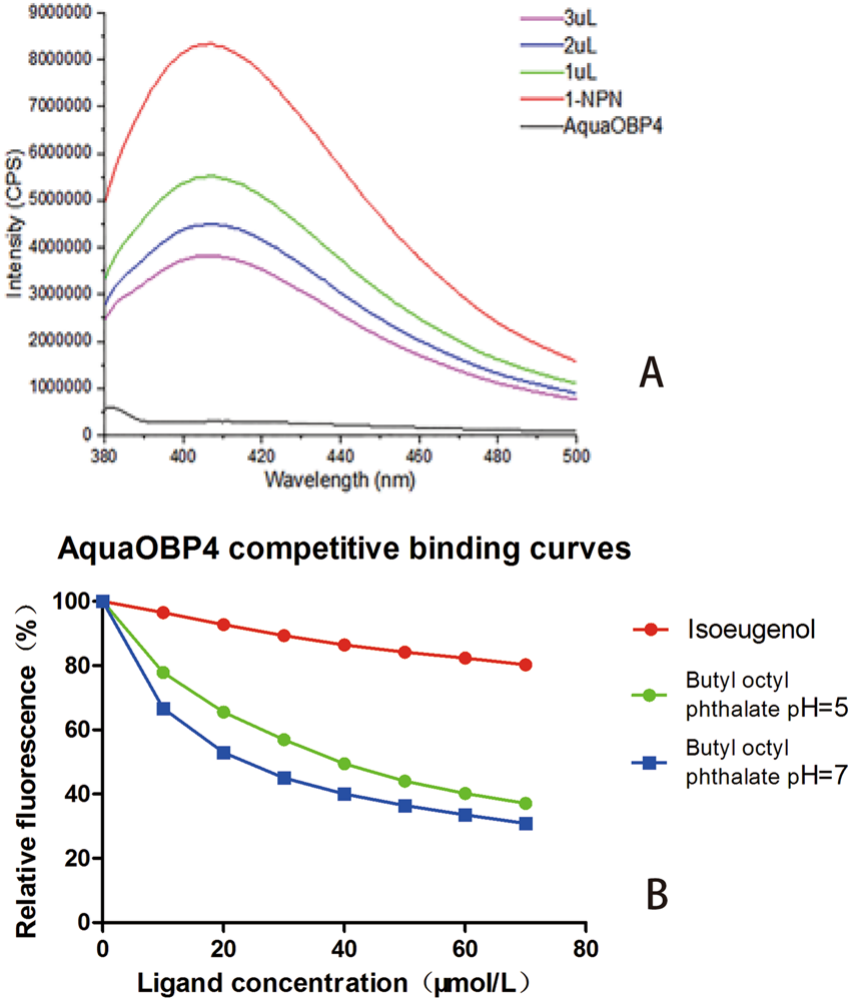
Binding curves of AquaOBP4 to the chosen compounds from the prediction model. (**A**) Fluorescence competition curves of butyl octyl phthalate to AquaOBP4 with the fluorescence reporter 1-NPN. The concentration of tested compound is 10 μM, and the final amount of 1-NPN is 2 μM. Ki = 12.7. (**B**) Binding curves of two compounds from the prediction model.

To further verify the prediction capacity of the 3D model of AquaOBP4, two new ligands, butyl octyl phthalate and isoeugenol, were selected based on their structural similarities and were also observed to dock in the same binding cavity of AquaOBP4. The docking results for butyl octyl phthalate showed the highest binding score among all ligands of 8.02. This ligand was also verified to exhibit the best binding capacity to AquaOBP4, with a Ki of 12.70 μM in the binding assay (Fig. 6). Surprisingly, one of the long ester chains of butyl octyl phthalate was located at the central Site 1, whereas another long ester chain stretched to Site 2, with an H-bond of 2.658 Å to Tyr77 in helix α3 of AquaOBP4. Butyl octyl phthalate clearly presented a better match with the whole binding cavity of AquaOBP4 and showed a better binding capacity to AquaOBP4. Furthermore, the docking score of isoeugenol was quite low, at only 3.86, and its binding conformation was positioned only in Site 2 of AquaOBP4. The binding affinity assay revealed a low Ki of 170.54 μM (Fig. 5). We also chose butyl octyl phthalate to perform a binding assay at pH 5. The Kd value of AquaOBP4 with 1-NPN was 1.488 ± 0.08 μM, and the Ki of butyl octyl phthalate was 16.81 μM at pH 5. Therefore, butyl octyl phthalate showed a lower binding affinity at pH 5 than at pH 7, suggesting that a lower pH could decrease its binding affinity for AquaOBP4 (Fig. 6).

### EAG and behavioral responses of binding ligands

**Figure 7:**
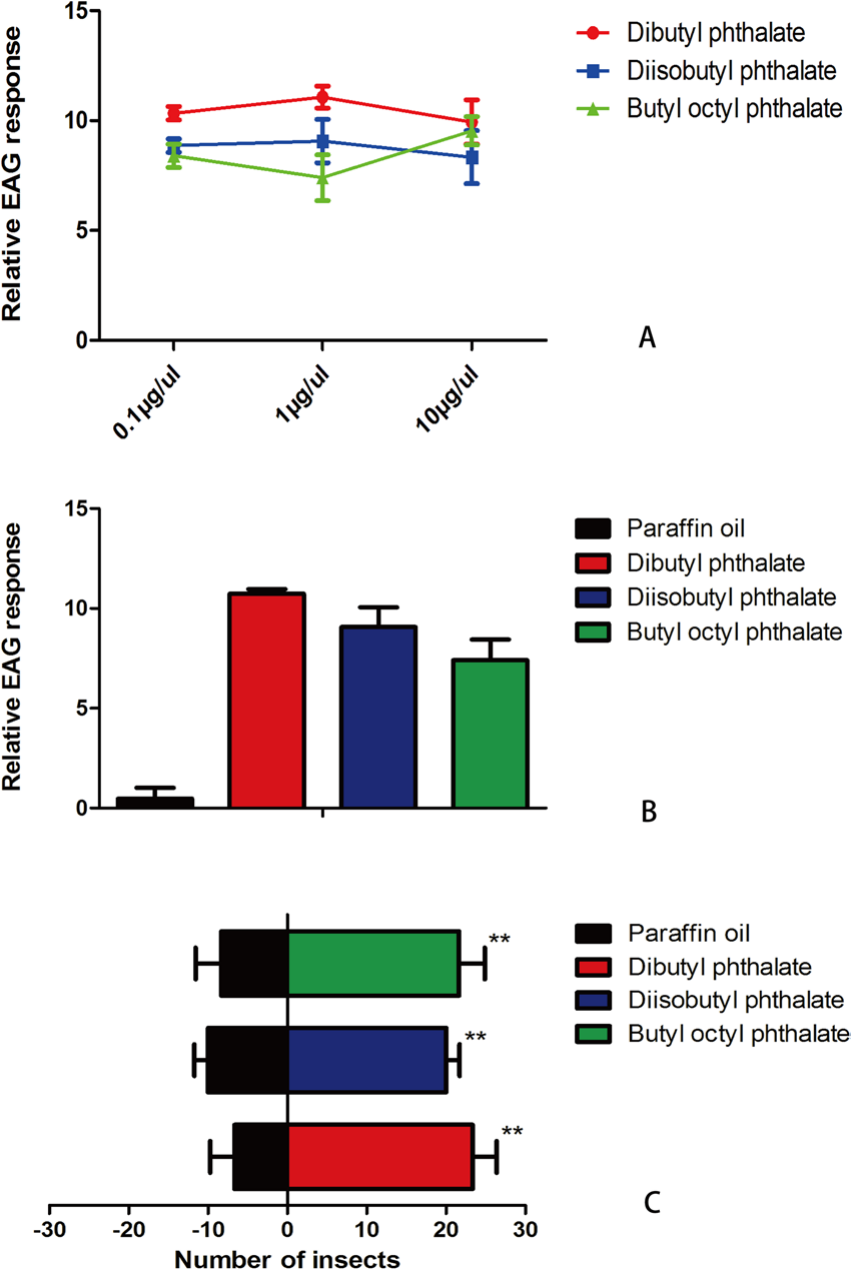
EAG and the behavioral responses of the ligands showing the best binding to AquaOBP4. (**A**) Dose-dependent curves of the tested ligands (0.1, 1 and 10 µg/µl). (**B**) Relative EAG responses of the three tested odorants at a concentration of 1 µg/µl. (**C**) Y-tube olfactometer results for the three tested odorants. The error bars indicate the SEM, and **indicates p < 0.01. N = 90.

To further test the biological functions of the three best ligands in terms of binding to AquaOBP4, EAG and Y-tube olfactometer tests were performed. Dibutyl phthalate, diisobutyl phthalate and butyl octyl phthalate all elicited strong EAG responses (Fig. 7B). However, no obvious changes in the responses were obtained when different concentrations of three ligands were tested (Fig. 7A), suggesting that even lower concentrations of these analogs could elicit saturated antennal responses. Additionally, no differences in the responses of the three ligands were observed in either the amplitude- or dose-dependent curves. The Y-tube olfactometer results revealed that the three tested odorants could all attract *A. quadriimpressum* adults (1 µg/µl) because the number of insects was significantly greater on the odorant side than on the control side (p < 0.01). Additionally, no large differences were observed among the three tested odorants, although dibutyl phthalate exhibited slightly higher attractiveness (Fig. 7C).

## Discussion

The fluorescence binding results showed that AquaOBP4 could easily bind phthalic acid derivatives, and the top three binding compounds all contained phthalic acid groups. However, AquaOBP4 did not bind to most phenylacetic acid single-lipid derivatives, such as methyl salicylate, methyl benzoate, acetic acid, and phenyl ester, with one exception, cis-3-hexenyl benzoate. No strict rules were observed, and only the references from the chemical structures themselves were used. Furthermore, AquaOBP4 showed no binding to methyl benzoate or leaf acetate, which are the precursors in the production of cis-3-hexenyl benzoate, but the binding of AquaOBP4 to cis-3-hexenyl benzoate suggested that the OBP-odorant complex was not merely a simple interaction between a specific chemical group and an amino acid residue; rather, the entire structure of the compound interacted with the conformation of OBP to influence the binding dynamics. Additionally, dibutyl phthalate and diisobutyl phthalate are isomers that show approximately the same binding affinity to AquaOBP4; some PRs have been reported to have the capacity to distinguish isomers^34,35^. Our results suggested that the selectivity of AquaOBP4 was not very strict, and the discriminate isomers were not initiated with a single OBP, which relies more upon ORs or higher olfactory neural pathways. Finally, butyl octyl phthalate is the analog of the isomers dibutyl phthalate and diisobutyl phthalate, although butyl octyl phthalate has four more carbons in one of its carbon chains than the two analogs. However, much stronger binding to AquaOBP4 was observed, indicating that the lengths of the carbon chains around analog chemicals could have an enormous impact on the binding affinity of AquaOBP4. Furthermore, a fluorescence binding assay conducted at pH 5 showed that a lower pH decreases the binding affinity of AquaOBP4, possibly due to a conformational change of AquaOBP4. This result has already been confirmed by structural studies in other insect species^13,18^. Most of the tested compounds did not bind to AquaOBP4 in the first round of tests, with only five ligands showing weak binding. Compared with the binding affinities of OBPs from other insect taxa, such as CquiOBP1 and AgamOBP1, the binding affinity of AquaOBP4 is relatively low^9,13^, and the results suggested that the best ligands for AquaOBP4 might not yet have been identified at this stage. Therefore, we decided to establish a reliable homologous model of AquaOBP4 for predicting the active odorants.

Molecular modeling has become one of the most important methods for studying the binding affinities between bioactive molecules and bio-macromolecules. To better understand the binding conformations of different ligands and AquaOBP4, the ligand–putative AquaOBP4 protein complexes were investigated through molecular docking. Two different sites in the binding cavity of the AquaOBP4 model were identified, and these ligand binding sites in the AquaOBP4 modeling were markedly different from the alcohol group from helix α3, helix α6 and the C-terminal strand in the LUSH template^36^. Additionally, the Trp60 residue of AquaOBP4 was observed to be important for the location and orientation of these ligands, whereas in the template of DmelOBP LUSH with alcohol, a group of the Thr57, Thr52 and Thr48 residues in helix α3 has been reported to form a network of concerted hydrogen bonds between the protein and the alcohol to provide a structural motif to increase the alcohol binding affinity at this site^36,37^. Invertebrate OBPs generally show a low isoelectric point, but AquaOBP4 is an alkaline protein, which caused us more difficulty in expressing and purifying this protein. As an alkaline OBP, DmelOBP LUSH has been better studied from both neurologic and biochemical aspects. In the present study, we expressed and purified another alkaline protein in a species unrelated to *Drosophila*. Binding assay and molecular docking results showed that although AquaOBP4 exhibited a high similarity to DmelOBP LUSH at the amino acid level, the selectivity and binding pocket of AquaOBP4 were quite different from those of DmelOBP LUSH^38^, which might indicate completely different biofunctionalities of these two OBPs.

Notably, AquaOBP4 also showed weak binding to cinnamaldehyde (79.26 μM), which is an antifeedant against *A. quadriimpressum*. Our previous results showed that cinnamaldehyde could elicit a strong physiological response on the antennae of *A. quadriimpressum*, and further behavioral tests confirmed the antifeedant function of cinnamaldehyde. This suggest that AquaOBP4 might participates in helping to transport cinnamaldehyde to olfactory receptors at the molecular level, however, it need more experiments to confirm such as by using RNAi technique, for that CSPs could participate in the transport or it could be transported in an unbound state (cinnamaldehyde is slightly soluble in aqueous solutions). Both β-ionone (Ki = 30.06 μM) and cis-3-hexenyl benzoate (Ki = 35.00 μM), which are proteins from the feces of adult *A. quadriimpressum*, also showed binding to AquaOBP4. Additional experiments are required to investigate the biological functions of these two odorants. The compound used for modeling validation, isoeugenol, showed a very low binding affinity to AquaOBP4, with a Ki of 170.54 μM, whereas another predicted compound, butyl octyl phthalate, showed the best binding affinity to AquaOBP4 detected to date, with a Ki of 12.70 μM. The results of fluorescence binding assays for the two new ligands coincided well with our docking model because the long ester chain of butyl octyl phthalate was located in the central Site 1, whereas another long ester chain stretched to Site 2. In addition, these results indicate that the predicted binding site of our docking model is reliable and can potentially be used in future large-scale compound screening for AquaOBP4. The top three binding compounds, dibutyl phthalate (Ki = 24.22 μM), diisobutyl phthalate (Ki = 26.77 μM) and butyl octyl phthalate (Ki = 12.70 μM), which originate from the leaves of elms, were selected for further electrophysiological and behavioral tests. Other studies have shown that these compounds also occur among the volatiles of some other plants, such as *Calycopteris floribunda* ^39^; these three odorants could have all elicited strong EAG responses and had significant attractive effects on adult *A. quadriimpressum*. Because AquaOBP4 also binds to cinnamaldehyde, which is an antifeedant, AquaOBP4 binding might be correlated with host specificity. Even at this stage, we still cannot firmly conclude that AquaOBP4 is not an aggregation pheromone-related OBP in *A. quadriimpressum*, similarly to *D. melanogaster*, because the aggregation pheromone component in *A. quadriimpressum* remains unclear, and the additional functions of AquaOBP4 require further exploration. However, our results suggest that the phthalic acid derivatives from elm leaves can be detected by *A. quadriimpressum* and can attract this pest via a mechanism that involves AquaOBP4. Additionally, AquaOBP4 is more likely linked with the foraging behavior of *A. quadriimpressum*, and the variety of functions of the insect OBP LUSH family is worth investigating in future studies.

## Electronic supplementary material

supplementary table S1

supplementary figure S1
