# Supplementary material for: Selectivity and ligand-based molecular modeling of an odorant-binding protein from the leaf beetle Ambrostoma quadriimpressum (Coleoptera: Chrysomelidae) in relation to habitat-related volatiles
Source: Sci Rep. 2017 Nov 13;7:15374. doi: 10.1038/s41598-017-15538-8 (PMC5684361; doi:10.1038/s41598-017-15538-8)
Supplement: Supplementary file 1 — supplementary table S1 [file 41598_2017_15538_MOESM1_ESM.pdf]

Selectivity and ligand-based molecular modeling of an odorant-binding protein from the leaf beetle *Ambrostoma quadriimpressum* (Coleoptera: Chrysomelidae) in relation to habitat-related volatiles

Yinliang Wang[1, 3], Yincan Jin[1, 3], Qi Chen[1, 3], Ming Wen[1, 3], Hanbo Zhao[1, 3], Hongxia Duan[2†] and Bingzhong Ren[1, 3*]

1 Jilin Provincial Key Laboratory of Animal Resource Conservation and Utilization, Northeast Normal University, Changchun, Jilin, China.

2 Department of Applied Chemistry, College of Science, China Agricultural University, Beijing, China.

3 Key Laboratory of Vegetation Ecology, MOE, Northeast Normal University, Changchun, China.

† Correspondence: Dr. Hongxia Duan

hxduan@cau.edu.cn

* Correspondence: Dr. Bingzhong Ren

bzren@163.com

Number	Name	Source	Relative quality(%) or peak area(×10^5)
1	1-(4-Ethylphenyl)-ethanone	Leaves of elm	1.09
2	methyl benzoate	Leaves of elm	0.77
3	cis-3-Hexen-1-ol	Leaves of elm	4.25
4	1-Methylnaphthalene	Leaves of elm	0.72
5	Caryophyllene	Leaves of elm	2.57
6	Dibutyl phthalate	Leaves of elm	35.09
7	Linalool	Leaves of elm	0.96
8	Nerolidol	Leaves of elm	0.81
9	Pentadecane	Leaves of elm	0.99
10	Tetradecane	Leaves of elm	0.53
11	Limonene	Leaves of elm	1.35
12	α-Farnesene	Leaves of elm	3.91
13	α- Pinene	Leaves of elm	10.15
14	1-Tridecene	Leaves of elm	0.99
15	Nonanal	Leaves of elm	6.14
16	Methyl salicylate	Leaves of elm	1.12
17	Dodecane	Leaves of elm	0.98
18	Benzaldehyde	Leaves of elm	0.86
19	trans-2-Hexenal	Leaves of elm	1.39
20	3,7-Dimethyl-1,3,6-octatriene，Ocimene	Leaves of elm	16.04
21	Diisobutyl adipate	Leaves of elm	0.99
22	Leaf acetate	Leaves of elm	
23	Diisobutyl phthalate	Leaves of elm	50.16
24	Benzyl benzoate	Leaves of elm	0.86
25	3',4'-Dimethylacetophenone	Leaves of elm	0.63

26	Phenylacetaldehyde	Cuticle of Larvae	
27	acetic acid, phenyl ester	Cuticle of Larvae	
28	hexanal	Phloem of elm trees	0.42
29	2-heptanone	Phloem of elm trees	0.58
30	myrcene	Phloem of elm trees	1.05
31	a-Terpinen	Phloem of elm trees	0.35
32	heptadecane	Phloem of elm trees	1.23
33	camphene	Adult feces	79.43
34	indole	Adult feces	718.81
35	(+)-cedrol	Adult feces	153.52
36	b-Ionone	Adult feces	216.66
37	cis-Jasmone	Adult feces	411.75
38	cis-3-Hexenyl benzoate	Adult feces	337.33
39	Ethyl palmitate	Adult feces	
40	Cinnamaldehyde	Oak trees	repellant
41	β-caryophyllene	Leaves of elm	0.76
42	1,2-benzenedicarboxylicacid,butyl octyl ester	Leaves of elm	0.15
43	n- hexadecane	Leaves of elm	0.55
44	2,4,6-tris(1,1-dimethylethyl)phenol	Leaves of elm	0.29
45	δ-cadinene	Leaves of elm	0.6
46	Butanedioic acid dibutyl ester	Leaves of elm	0.57
47	Pentanal-2-methy	Phloem of elm trees	0.34
48	Cis-2-peten-l-ol	Phloem of elm trees	0.24
49	1- methyl naphthalene	Phloem of elm trees	0.22
50	α-humulene	Phloem of elm	0.44

		trees	
51	Cis-β-ocimene	Phloem of elm trees	0.74
52	phenyl acetate	Adult feces	22.5
53	epoxylinalol	Adult feces	38.54
54	1-heptatriacotanol	Adult feces	68.16
55	linalool	Adult feces	134.69
56	Dipentene oxide	Adult feces	96.4
57	1-heptatriacotanol	Adult feces	68.16
58	Acetic acid, butyl ester	Adult feces	17.06
59	tmtt	Adult feces	159.75
60	isoarmadendrene epoxide	Adult feces	136.5
