# Supplementary material for: Selectivity and ligand-based molecular modeling of an odorant-binding protein from the leaf beetle Ambrostoma quadriimpressum (Coleoptera: Chrysomelidae) in relation to habitat-related volatiles
Source: Sci Rep. 2017 Nov 13;7:15374. doi: 10.1038/s41598-017-15538-8 (PMC5684361; doi:10.1038/s41598-017-15538-8)
Supplement: Supplementary file 2 — supplementary figure S1 [file 41598_2017_15538_MOESM2_ESM.pdf]

Selectivity and ligand-based molecular modeling of an odorant-binding protein from the leaf beetle *Ambrostoma quadriimpressum* (Coleoptera: Chrysomelidae) in relation to habitat-related volatiles

Yinliang Wang[1, 3], Yincan Jin[1, 3], Qi Chen[1, 3], Ming Wen[1, 3], Hanbo Zhao[1, 3], Hongxia Duan[2†] and Bingzhong Ren[1, 3*]

1 Jilin Provincial Key Laboratory of Animal Resource Conservation and Utilization, Northeast Normal University, Changchun, Jilin, China.
2 Department of Applied Chemistry, College of Science, China Agricultural University, Beijing, China.
3 Key Laboratory of Vegetation Ecology, MOE, Northeast Normal University, Changchun, China.
† Correspondence: Dr. Hongxia Duan
hxduan@cau.edu.cn
* Correspondence: Dr. Bingzhong Ren
bzren@163.com

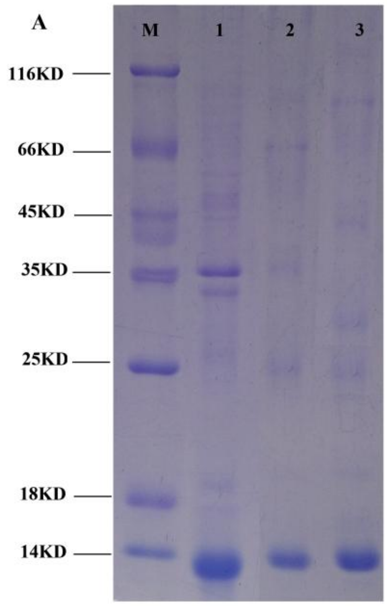

B

MRIMCKMNCTIIILVTSIILNVSGMNEKQMEAA
LKMVKNVCKPKTKATDADIDKMHKGDWNIDH
TAMCFILCALNMYKLMNTDNTLNYESALIQLKQ
LPDSFREPTRQCMENCKDAAVTLGDKCIAAYEL
AKCMYICNPEKFFLH

Fig S1. AquaOBP4 expression and purification. **A. SDS-Page results of purified AquaOBP4**, M: protein marker; 1. Protein with 8M urea; 2. Purified original protein with buffer A (20mM Tris-HCl pH8.0， 50mM NaCl， 0.1% TritonX-100， 100mMimidazole，8M urea); 3. Purified original protein with buffer A (20mM Tris-HCl pH8.0，50mM NaCl，0.1% TritonX-100，250mM imidazole，8M urea). **B. Amino acid sequence of AquaOBP4**
